# Quo Vadis, *Orthotrichum pulchellum*? A Journey of Epiphytic Moss across the European Continent

**DOI:** 10.3390/plants11202669

**Published:** 2022-10-11

**Authors:** Vítězslav Plášek, Lukáš Číhal, Frank Müller, Michał Smoczyk, Ivana Marková, Lucie Fialová

**Affiliations:** 1 Department of Biology & Ecology, University of Ostrava, Chittussiho 10, CZ-710 00 Ostrava, Czech Republic; 2 Institute of Biology, University of Opole, Oleska 48, PL-45-052 Opole, Poland; 3 Silesian Museum, Nádražní okruh 31, CZ-746 01 Opava, Czech Republic; 4 Institut für Botanik, Technische Universität Dresden, D-01062 Dresden, Germany; 5 Stanisław Staszic High School in Rzepin, Wojska Polskiego 30, PL-69-110 Rzepin, Poland; 6 Bohemian Switzerland National Park Administration, Pražská 52, CZ-407 46 Krásná Lípa, Czech Republic

**Keywords:** bryophytes, climate change, distribution, ecological requirements, epiphytic moss, expansion, Maxent, species distribution modeling

## Abstract

*Orthotrichum pulchellum* is a species of epiphytic moss in which a significant expansion from the oceanic part of Europe to the east of the continent has been observed in the recent two decades. The improvement in air quality in Central and Eastern Europe, but also climate change, probably plays a role in this. This study shows what direction of its spreading we can expect in the future. Ecological niche modeling (ENM) is a widespread method to find out species niches in environmental and geographical space, which allows us to highlight areas that have a higher probability of occurrences of the studied species, based on identifying similar environmental conditions to those already known. We also made predictions for different future scenarios (CMIP5 climatology datasets for the years 2041–2060). Because we were not able to distinguish between historical and newly settled areas, and so, had to use some of the traditional approaches when modeling invasive species, we proposed to use niche clusters based on environmental layers to split the data of all known occurrences and make models separately for each cluster. This approach seems reasonable from the ecological species point of view because using all the morphologically same samples could be misleading. Altogether, 2712 samples were used from three separate niche clusters. For building the models, the Maxent algorithm was used as a well-tested, well-accepted, and commonly used method.

## 1. Introduction

The epiphytic moss *Orthotrichum pulchellum* Brunt. is evaluated as an oceanic species with a disjunct distribution including oceanic zones of western North America and western Europe [1,2,3,4,5]. On the European continent, it was reported from many countries situated mainly along the western seacoasts—from southern Scandinavia to northern Spain [6,7,8]. It has also been reported from Sicily and continental Italy [9]. Furthermore, the species is also known from very few and scattered localities in SE Europe (Bosnia-Herzegovina [10], Slovenia [11], and Serbia, [12]). Records from Crete are classified as doubtful by Hodgetts and Lockhart [13]. From the west of the Atlantic coast, the distribution area of the species recently extends to the Central European countries. There are known to be a number of 46 of its localities also in Germany, and the results of the field research suggest that the moss is spreading to the east [14,15]. For example, in Saxony, the species was found in 2002 for the first time, and after two years, it was observed in more than 20 localities [16] and recently more than 600 records were documented. In the Czech Republic, the species was recorded for the first time in 2006 [17,18], and within the next few years, about 40 new localities were known there, situated mainly in the western part of the country [19]. The first record of *O. pulchellum* from Poland does originate from the 19th century [20], but subsequently, it was found only 145 years later in the country [21]. At that time, several additional sites, situated in the western, southern, and central parts of the country, were found, and the number of its locations increased there [22,23,24].

The spreading of this oceanic species inland is apparently related to both the climate changes and air quality improvements [25,26,27,28]. Among the significant climate elements with an influence on the expansion can be considered the effect of mild winter temperatures and possibly also higher precipitation. Such climate changes have recently been confirmed by meteorologists in Europe [29]. These changes are recognized as a significant pressure on nature and biodiversity and a major driver of ecosystem transformations [30].

## 2. Materials and Methods

### 2.1. Species Identifying

**Figure 1:**
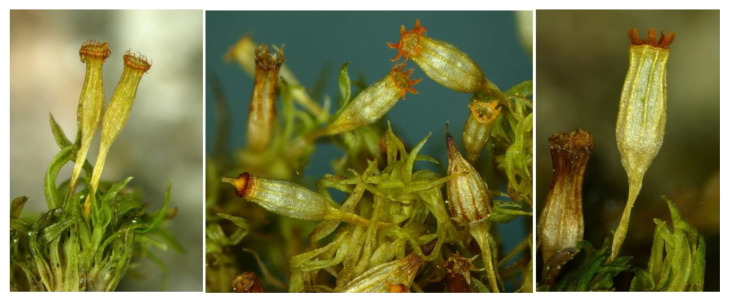
*Orthotrichum pulchellum*—Habit of the fertile plants. All taken from specimen collected by V. Plášek in Krušné hory Mts, Czech Republic, 13 June 2019 (#OSTR-3245).

*Orthotrichum pulchellum* is an epiphytic moss, belonging to the family Orthotrichaceae [31,32,33]. Plants are about 1–2 cm high. From most related Central European orthotrichaceous species, it is distinguished mainly by crisped-flexuose stem leaves and exserted capsules. These characteristics make it similar to the species of the genus *Ulota*, with which it may be confused. However, *O. pulchellum* can be easily distinguished from them by having 16 conspicuously orange exostome teeth, rarely united into 8 pairs, immersed stomata, and at the base, a glabrous, more or less plicate calyptra (Figure 1). In addition, the marginal cells of the leaf base (thin-walled, hyaline, quadratic), a marked characteristic for *Ulota* species, are lacking in *O. pulchellum*.

### 2.2. Species Occurrence Data

Occurrence data were downloaded from the GBIF (Global Biodiversity Information Facility http://www.gbif.org, accessed on 1 May 2022), and obtained from the bryological collections (DR, KRAM, OP, OSTR, private collections of M. Smoczyk, S. Rosadziński, and I. Marková). Because the first mentioned often includes large sampling bias and errors, which may reduce the ability to accurately project the distributions of invasive plants [34], we used a set of different approaches of CoordinateCleaner [35] to clean the data. First, we used an automatic cleaning algorithm that helped us to remove errors that are common to biological collections, including sea coordinates, zero coordinates, coordinates assigned to country and province centroids, outlier coordinates, and coordinates assigned to biodiversity institutions. We also removed coordinates with uncertainty higher than 1 km and records with suspicious individual counts lower than 0 and higher than 99. As the last step of the procedure, we checked the data for coordinate conversion errors based on the misinterpretation of the degree sign (°) as a decimal delimiter and also the occurrence of records derived from rasterized collection designs or subjected to strong decimal rounding. After visualization of the diagnostic output, we could say that the dataset did not show rasterized collection schemes. We used only data from the years between 1979 and 2020 (including) to correspond to the used environmental layers time span (near current conditions 1979–2013) and also included as much as possible from the recent data. To reduce spatial autocorrelation and sampling bias, we decided to use the ntbox package [36] and filter occurrences by threshold distance for duplicates by (δ) = 0.05 parameter. Altogether, this gave us 2712 samples for the next analysis.

Usually, there are three ways to use occurrences in the prediction of invasion of a species: first, to use natural and invasive data; second, to use only natural data; and, third, to use only invasive data, which all have their advantages and weaknesses. Probably, the most straightforward approach would be to use all the available data and make a global prediction of the studied species to capture most of the species niche, but such a prediction of the niche does not account for the specificities of local adventive ranges. This could be partly solved by also making a regional prediction weighted by global prediction [37], but even weighted prediction was not an ideal solution in our case, because the global model strongly underestimated populations in Central Europe. Moreover, the use of natural data only was not possible at first, because we were not sure of the natural area of the studied species and, second, because of the high rate of nonhomologous environmental conditions between the continental Europe and the rest of the studied area.

So, as we were mostly interested in the prediction of the newly spreading populations of the studied species in continental Europe in 30 sec resolution, we decided to focus on the occurrences from this area; on the other hand, we also wanted to capture as much as possible of the niche suitable for these conditions for modeling the species. To deal with this problem, we defined the studied species, not by its geographical area in Central Europe, but, at first, by its position in environmental space. Reselection of the samples in the geographical space for further analysis was possible due to Hutchinson’s duality (the duality between “niche” and “biotope” proposed by G. Evelyn Hutchinson). For this purpose, we built its niche from all available data and then split the data based on their environmental requirements. For this purpose, we used niche clusters built by the niche clustering function by ntbox [36] to characterize the niche based on environmental layers and then used the cluster data separately. That seemed reasonable because we were not using all morphologically same occurrences without any further questioning about their position in environmental space. With this approach, we combined the morphological conception of the species to define the species, which actually could be adapted to different conditions, and also the ecological conception of the species, which looks like a better solution, because using morphologically same samples with adaptations to different conditions without any further questioning about their environmental demands could be even considered as modeling them as morphologically same, but in the view of the ecological conception of the species as different species in one model.

### 2.3. Environmental Layers and Variable Selection

We used a high-resolution, global dataset designed for ENM applications, Chelsa (climatologies at high resolution for the earth’s land surface areas) data [38] in a horizontal resolution of 30 arcsec (~1 km) which was built to provide free access to high-resolution climate data for research and application and is constantly updated and refined. From the initial set of 19 variables, we removed the annual mean temperature (bio01) and the annual precipitation (bio12) because factors such as average temperatures and precipitations may have little biological meaning [39], and limiting variables have a higher biological meaning to the distribution of the species [40,41]. We also removed the mean temperature of the wettest quarter, mean temperature of driest quarter, precipitation of the warmest quarter, and precipitation of the coldest quarter (bio08–09, bio18–19) as these variables are commonly excluded from the pool of selected variables to build a model, due to a number of apparent artefacts [42,43]. To reduce dimensionality and to reduce collinearity among layers, we applied principal components analysis to the remaining bioclimatic layers. We removed layers strongly correlated according to a correlation threshold of 0.85. Seven environmental layers were included (bio02, bio03, bio04, bio06, bio10, bio13, and bio15). From this set of variables were made eight different sets of environmental layers with at least six layers per set built by the kuenm_varcomb function of the KUENM package [44] for further testing.

### 2.4. Future Scenarios

To project models to the future scenarios, downscaled CMIP5 climatology datasets for the years 2041–2060 were used for mean monthly maximum temperatures, mean monthly minimum temperatures, monthly precipitation amounts, and several derived parameters. The downscaled data was produced using climatologically aided interpolation based on the 1979–2013 reference climatologies from the CHELSA dataset [38]. In selecting CMIP5 scenarios, we followed the recommendations provided when obtaining future Chelsa data scenarios (https://chelsa-climate.org/future/, accessed on 1 May 2022) and chose 5 models to represent the decent amount of uncertainty in climate model projections. To select specific scenarios with the lowest amount of interdependence, we followed the guidelines in Sanderson [45] and selected ACCESS1, CESM1-BGC, CMCC-CM, MIROC5, and MPI-ESM-MR scenarios with two RCPs (representative concentration pathways): RCP 4.5 and RCP 8.5.

### 2.5. Background Points and Background Area

We followed Phillips and Dudík [46] and included 10,000 random background points to characterize the ‘background’ of environments available to the species from a background area. In order to restrict the choice of background points to realistically reachable locations from which the background points were taken, we used 50 km circular buffers around presence points. Because this area should be based on dispersal capacity and the history of the species and should not include small areas too close to presence points or large regions that the species does not inhabit, we also included in buffer areas only areas of ecoregions in which at least one sample was present. Areas of ecoregions without any sample points were excluded from background areas. This was set because, in general, the boundaries of these regions correspond to shared sets of distributional limits of species across landscapes, which may be informative about barriers that have repeatedly constrained the distributional potential of species [47]. Buffers and selections of ecoregions were made for each cluster separately to correspond to the specific demands of each cluster.

### 2.6. Used Algorithm

For building models, we used the Maxent (Version 3.4.4) algorithm [48]. Maxent is a machine-learning method that calculates a raw probability value for each pixel of the study region [46,49]. Among many of the species distribution model algorithms, Maxent is a well-tested, well-accepted, and commonly used method, and it has been shown to be a top performing algorithm compared to other methods [50,51,52].

### 2.7. Feature Class and Regularization

According to the feature types and settings called “regularization parameters”, we could control the complexity of dependencies [46]. This procedure was realized based on the description of the model calibration and selection process on the final report of the ku_enm_ceval function implemented in the kuenm R package [44]. All in all, 280 candidate models, with parameters reflecting all combinations of 7 regularization multiplier settings (0.1, 0.4, 0.7, 1, 2, 4, 7), 5 feature class combinations (“l”, “lq”, “lqp”, “lqpt”, “lqpth”), and 8 distinct sets of environmental variables were evaluated for each cluster. Model performance was evaluated based on statistical significance (Partial_ROC), omission rates (OR), and the Akaike information criterion (AIC). A selection process was made for each cluster data separately.

### 2.8. Extrapolation Risk

Models were built using a background area and then reprojected onto the area of the European continent, but because projection into different geographical areas than those where the model was calibrated involve projection into novel covariate space, we had to analyze the extrapolation risk of the chosen environmental layers. This was realized by the ExDet tool, based on the Mahalanobis distance [53], which measures the similarity between reference and projection domains. With this tool, we were able to visualize novel combinations between covariates and reveal types of novelties in reprojected areas. Areas that showed an extrapolation risk of NT1-type novelties (areas in the projection space with at least one climate covariate outside its range) were also included in the model but should be evaluated with caution. Additionally, NT2-type novelties (indicating areas that were within the univariate coverage of the reference data, but represent nonanalogous covariate combinations) were evaluated, but after visualization, the effect of this mistake (NT2 type) was negligible. Areas that did not show either NT1 or NT2 novelties were similar to the reference data and fell within the range of reference covariates, and so, captured the same covariate combinations [53].

### 2.9. Model Settings and Evaluation

**Figure 2:**
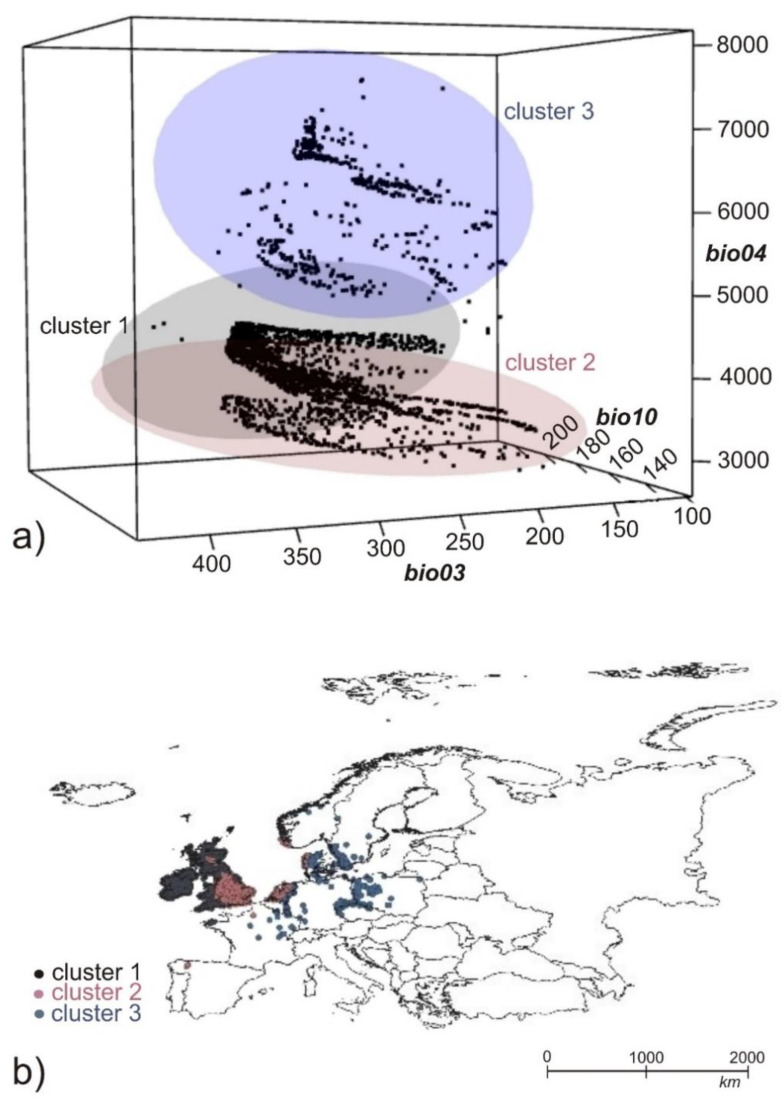
Visualization of the three clusters in the environmental space (**a**) based on the first three environmental layers (bio04, bio10, bio03) with strong visual differentiation along with bio04 layer and their representation in the geographical space (**b**).

For calculating individual models and preparation of the data, R (R Development Core Team 2020) studio was used, and Qgis [54], for occurrence data preparation and environmental layers, was used. For building the models, each cluster of data was split into 70% of the initial data points and then evaluated on the remaining 30% of data. Variables in Table 1 were used for building the final models. Three different clusters of data based on the niche K clustering from the ntbox package [36] were used separately for building the models. We chose this number of clusters after visualization of 2, 3, 4, and 5 clusters, from which splitting into three clusters seemed reasonable to catch the diversity between our data in environmental and geographical space and also to use as much from the data as possible for each separate model. After visualization of the clusters in environmental space, we were able to visualize those clusters in geographical space and to use those occurrences for further modeling approaches. The approximate expansion area for cluster 1 was England, Netherlands, Denmark, Norway, and Spain; for cluster 2, Scotland, Ireland, and the west and north coast of England; and for cluster 3, France, Netherlands, Germany, Belgium, Norway, Sweden, Poland, and Czech Republic (Figure 2).

For each cluster of data, five CMIP5 climatologies datasets for the years 2041–2060 with two RCPs and one reprojection to Europe with near current conditions (1979–2013) were used. The best criteria for each model were evaluated with the kuenm_ceval function from the KUENM package [44], and the final models were built. As the best feature class and regularization multiplier for each cluster were evaluated, the combinations are given in Table 2. For building the final model, we used the kuenm_mod function of the KUENM package [44] and as the settings, we chose to run 10 iterations of the 10-fold cross-validation with extrapolation and clamping as the type of projections [55]. The average output of replicates was used to visualize the final models. For evaluation of the models, we used AUC with the following categories of AUC: invalid (<0.6), poor (0.6–0.7), fair (0.7–0.8), good (0.8–0.9), and excellent (0.9–1.0) [56].

## 3. Results

### 3.1. Clusters Distribution along Environmental Gradients

Using the analysis, we managed to divide all the samples of the studied species into three clusters, which within the environmental space were mostly divided along the environmental gradient bio04 (temperature seasonality—standard deviation of the monthly mean temperatures). This variable means that in its lower values the environment is thermally more balanced and there is no greater temperature fluctuation within individual seasons, while at the opposite end (in higher values of this variable), temperature fluctuations are higher between seasons. After visual inspection of the results presented in Figure 2a, it was visible that the first two clusters were found in the lower values of this variable, while cluster 3 occurred in the higher values and also had a greater dispersion within the scale.

So, what does this mean for our cluster division? That we have, on the one hand, two clusters where the studied species grows in relatively stable temperature conditions (oceanic climatic zone) and does not spread, and on the other hand, one cluster that grows in seasonal temperatures that vary much more (continental climatic zone) and also, unlike the first two clusters, further spreads and can obviously survive in a larger range of environmental temperature conditions.

### 3.2. Model Predictions for Each Cluster

Cluster 1 did not show any suitable conditions outside its already known areas in the near current conditions. This also confirms that the use of this cluster for modeling niche in continental Europe will be actually giving us no additional information for such a model (same as for cluster 2). In the face of changing climate conditions, we can see that species are losing their current conditions even in the areas where they are now abundant. As the trend of the future expansion/shrinking, we can see the moving of suitable conditions to northern Europe, but the prediction is occurring in the lower scale of values.

Cluster 2 shows suitable conditions for near current conditions outside its known areas only in the west coast of France and also in Iceland with a few other areas with very low scale values in other parts of Europe, but what is more interesting is that when reprojected to the future scenarios, maps did not show any suitable conditions, not even on areas already known. That means it can be assumed that populations of this cluster could even disappear if they do not adapt to the changing conditions. Those future scenario maps are not displayed in this study, because they include only zero values (no climatic suitability).

**Figure 3:**
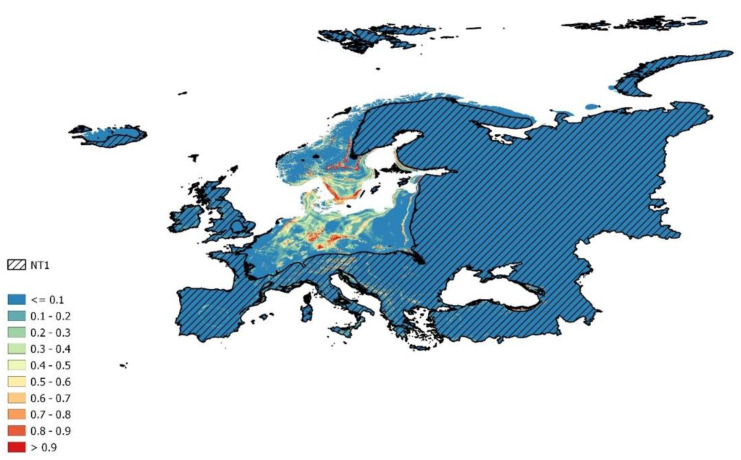
Climatically suitable areas on the scale from 0 to 1 (blue = low, red = high) for cluster 3 data reprojected to near current (1979–2013) conditions in Europe with displayed NT1 areas (hatched).

**Figure 4:**
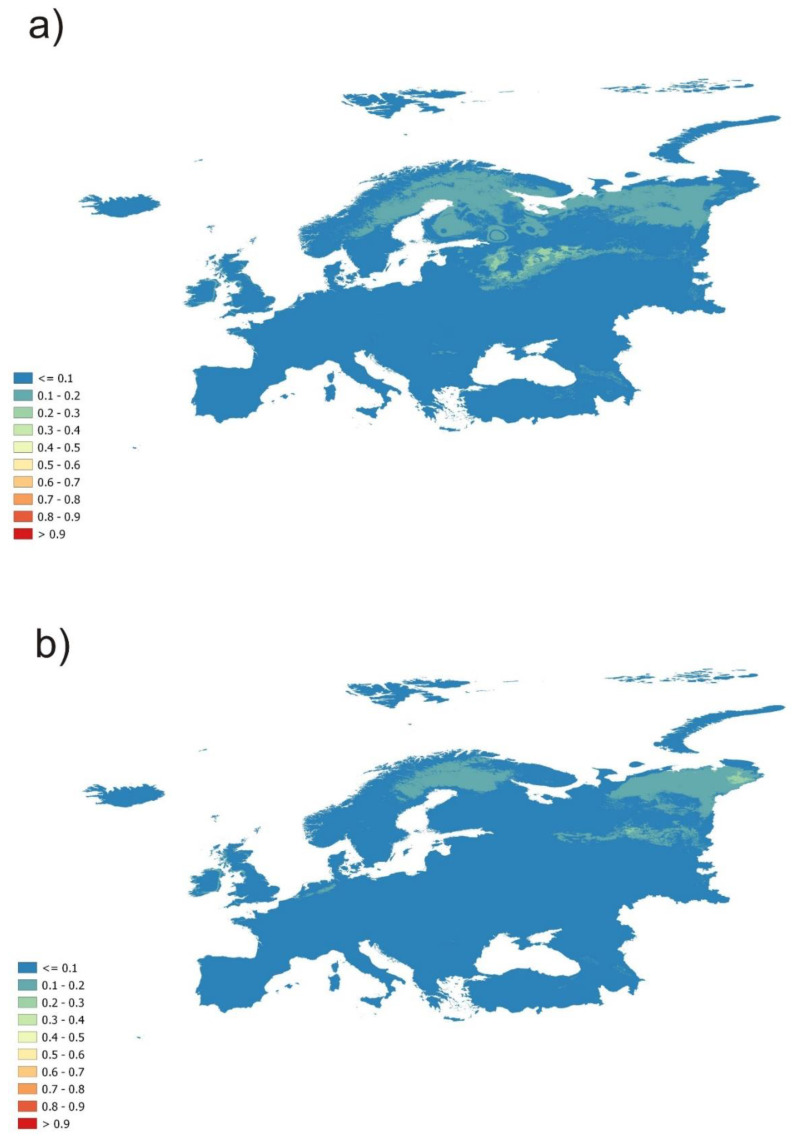
Climatically suitable areas on the scale from 0 to 1 (blue = low, red = high) for cluster 3 data reprojected to future conditions of CMIP5 climatology datasets in Europe: (**a**) ACCESS1 RCP 4.5, (**b**) ACCESS1 RCP 8.5 (two representative concentration pathways).

Cluster 3, on the other hand, provides us with a good image of the suitable habitats in homologous parts of continental Europe (and also in the nonhomologous parts which should be evaluated with caution) (Figure 3). This cluster was best suited for our research and, therefore, we also present its geographical visualization, unlike the first two mentioned clusters which achieved either similar results, as in the case of the first cluster, or extinction, as in the case of cluster 2. This prediction also shows areas not recorded yet as suitable for the studied species (red areas in Figure 3) and so, should be aimed at field research focused on the studied species. Those areas are concentrated in Germany, Sweden, south France, and, interestingly, also around the eastern part of the Black Sea. As for the parts with higher prediction rates but inside nonhomologous areas, we can point out northern Italy, Austria, or the Balkan states. Future predictions of this cluster show an interesting trend of disappearing of the population in Central Europe and moving to the northeast parts of Europe (Figure 4). As the trend of the future expansion/shrinking, we can see, once again, the moving of suitable conditions to northern Europe, but the prediction is occurring in the lower scale of values. From those results, it seems that known populations could disappear from the recent localities unless they adapt to new conditions, which seems to be more presumable. Additionally, because of the mentioned results of cluster distribution along environmental gradients, cluster 3 can survive in a larger range of environmental temperature conditions. For geographical visualization, we chose only the first scenario from the guidelines in Sanderson et al. [45], ACCESS1 with two representative concentration pathways, but all future scenarios showed a similar trend of disappearing from Central Europe and moving to its northern parts.

## 4. Discussion

Although there is relatively limited evidence of current extinctions caused by climate change, studies suggest that climate change could surpass habitat destruction as the greatest global threat to biodiversity over the next few decades [57]. The extent to which species can balance out the loss of suitable habitats due to climate change by shifting their ranges is unclear. Moreover, an important question is if the bryophytes—although they are organisms with highly efficient wind-dispersal—can keep up with projected changes in their areas of suitable climate [58].

In our study, we presented a new approach for studying and building environmental niche models for the expansive species of *Orthotrichum pulchellum* as a fast-spreading species over continental Europe. We decided to separate the known populations into separate clusters based on their position in the environmental space due to the different ecological requirements of European populations. Such a concept seems reasonable, as we can distinguish populations not by their geographical position, such as in the case of using natural vs. invasive areas data, but based on their environmental similarity and, due to Hutchinson’s duality, can specify separate geographical areas for further analysis. With this approach, we were able to not include areas and samples which would be not able to reach environmental conditions in the studied area and could be even considered as separate ecotype-different groups.

If we follow the chronology of the spread of our model species, *Orthotrichum pulchellum*, we see that the gradual onset of climatic changes at the end of the last century significantly helped this epiphytic moss to expand its range, especially from western to Central and Eastern Europe. The favorable influence of warmer winter periods erased the differences between winter and summer, which were previously completely different in terms of temperature and humidity. This opened the way for the species to migrate eastwards to the more continental part of Europe for this oceanic species. However, now that climate changes are manifesting with increasing intensity, their consequences, which were previously very favorable for the spread and establishment of the species, are gradually becoming more and more limiting.

The results presented in the study showed that in the species *Orthotrichum pulchellum* we can observe three ecotype-different groups. Each of them has slightly different ecological requirements, and each of them will, therefore, react differently to expected changes in environmental conditions caused by climate change. For clusters 1 and 2, the model indicates a high probability of endangerment of present populations by changing conditions. To persist, individuals, populations, or species must produce adaptive responses. These may involve intraspecific variation in morphological, physiological, or ecological traits, which can occur on different time scales within the populations’ spatial range [59]. However, one of the crucial questions in the debate on ecological effects of climate change is whether or not species will be able to adapt fast enough to keep up with the rapid pace of changing climate [60,61,62]. If this does not happen, then the model predicts that most of the populations of clusters 1 and 2 will be severely threatened or extinct.

However, we paid the most attention to “cluster 3”, as its populations occur mainly in the territory of Central Europe, where the fastest documented spread of species is currently taking place. According to the results of the modeling, the populations of this cluster will continue to expand their range in the future to the central and eastern parts of Europe, but also to the north and south under the near current conditions. They are not in any serious threat.

As already mentioned, important factors for the spreading seem to be mainly mild winter temperatures. As well, the humidity is also important. In general, the annual precipitation is constant in Central Europe, but the oceanic species also require higher humidity, e.g., in Saxony most records are situated in mountainous areas with, in general, higher precipitation. Records at lower elevations are mostly situated in narrow valleys along streams with locally humid site conditions [16,63]. The situation is similar in Poland, where most of the records were registered in the southern part of the country (the Sudety mountain range), in mountainous areas with higher precipitation. Similarly, most of the records from the western part of Poland (Lubuskie Lakeland) come from the valleys of small rivers, stream sides, or lakeshores characterized by higher humidity conditions [22,23,24].

A sign that the species has adapted well to the site conditions in the newly colonized Central European area is that it is very variable with regard to the colonized wood species. In the new area in Saxony, the species was found on 10 woody genera (a total of 12 woody species), including some that are normally rarely or not colonized by epiphytes. Finds on *Larix decidua*, on which the species has been identified three times, are particularly noteworthy. An even more pronounced spectrum of phorophytes inhabited by *Orthotrichum pulchellum* is in Poland, where the species was found on 16 woody genera (a total of 19 woody species). This testifies to the ability of this species to adapt well to new environmental conditions; therefore, we assume that even though the bioclimatic conditions will not be suitable for this species in the future, it will not die out, and it will be able to adapt to new conditions, but this will be absolutely necessary, if the climate scenarios we used for this study come true, there will be a drastic decrease in locations with conditions suitable for the occurrence of this species based on its current occurrence.

Furthering our understanding of climate change gives an important insight into what responses we can expect in the future, yet the magnitude of impacts on biota caused by anthropogenic climate change remains difficult to predict [64]. New research is constantly improving our understanding of the responses of species to climate change, yet often reminds us that current knowledge is inadequate, and that predictions of future impacts are fraught with uncertainty. It is important to improve predictions of climate-change impacts on biodiversity. They are similar to the ones that were used by us.

Besides *Orthotrichum pulchellum*, in the last three decades, several oceanic-Mediterranean epiphytic bryophyte species were recorded in new parts of Central Europe influenced by the improving air quality and/or global warming. The statements made for *Orthotrichum pulchellum* are valid in the same or a similar way for the spreading behavior of these species. Examples of such species are *Cryphaea heteromalla* (Hedw.) D.Mohr (first recorded in Saxony in 2009 [65,66]; first recorded in Poland in 2017 [67]; first recorded in Czech Republic in 2018 [68]), *Lewinskya acuminata* (H.Philib.) F.Lara, Garilleti and Goffinet (first recorded in Germany in 2003 [69]; first recorded in the Czech Republic in 2019 [70]), *Ulota phyllantha* Brid. (first recorded in Saxony in 2013 [71]), and *Zygodon conoideus* (Dicks.) Hook. and Taylor (first recorded in Saxony in 2008 [66]).

## Figures and Tables

**Table 1:** Environmental layers used to build Maxent models after layers were removed in the preselection steps and also strongly correlated layers according to a correlation threshold of 0.85.

Environmental Variables:
bio02 = Mean Diurnal Range
bio03 = Isothermality
bio04 = Temperature Seasonality
bio06 = Min Temperature of Coldest Month
bio10 = Mean Temperature of Warmest Quarter
bio13 = Precipitation of Wettest Month
bio15 = Precipitation Seasonality

**Table 2:** Calibration results of different clusters based on kuenm_ceval function of KUENM package with the number of samples in each cluster and the mean AUC score of the final models.

Cluster No.	No. of Samples	Environmental Layers Used *	Feature Class Combinations	Regularization Multiplier	Mean AUC Score
1	925	bio04, bio10, bio03, bio15	lqpth	2	0.733
2	1368	bio04, bio10, bio02	lqpt	0.7	0.743
3	419	bio04, bio10, bio03, bio15, bio06, bio02	lqpth	0.1	0.786

* Layers are in descending order by their percent contribution to the model (only layers with contribution >5 % were used for final models).

## Data Availability

Not applicable.
